# Knowledge, barriers, and facilitators for promoting cardiovascular health in a Latino community: a qualitative sub-study of the Skills-based Educational Strategies for Reduction of Vascular Events in Orange County

**DOI:** 10.3389/fpubh.2025.1531775

**Published:** 2025-06-18

**Authors:** Bernadette Boden-Albala, Darnisha Draughter-Espinoza, Megan Castro, Desiree Gutierrez, Cassandra Cardenas, Matthew J. Landry, Jeffrey Wing, Bruce Albala

**Affiliations:** ^1^Department of Health, Society, and Behavior, Joe C. Wen School of Population & Public Health, University of California, Irvine, Irvine, CA, United States; ^2^Department of Epidemiology & Biostatistics, Joe C. Wen School of Population & Public Health, University of California, Irvine, Irvine, CA, United States; ^3^Department of Neurology, School of Medicine, University of California, Irvine, Irvine, CA, United States; ^4^Department of Population Health and Disease Prevention, Joe C. Wen School of Population & Public Health, University of California, Irvine, Irvine, CA, United States; ^5^Division of Epidemiology, College of Public Health, The Ohio State University, Columbus, OH, United States; ^6^Department of Environmental and Occupational Health, Joe C. Wen School of Population & Public Health, University of California, Irvine, Irvine, CA, United States; ^7^Department of Pharmaceutical Sciences, School of Pharmacy and Pharmaceutical Sciences, University of California, Irvine, Irvine, CA, United States

**Keywords:** community participation, community-institutional relations, focus groups, family, social determinants of health, cardiovascular disease, health inequities

## Abstract

**Introduction:**

Cardiovascular disease (CVD) is the leading cause of death, and disproportionately affects racial-ethnic groups. Community-engaged research is an important avenue to address health disparities, and understand barriers faced by vulnerable populations. This qualitative study is a sub-study of the Skills-based Educational Strategies for Reduction of Vascular Events in Orange County (SERVE OC) clinical trial (Trial ID NCT05641519), which employed focus group discussions to gain insight into community understanding of CVD within the local Latino community of Orange County. The study also aimed to identify themes of (1) community knowledge, beliefs, and attitudes toward CVD prevention, (2) barriers and facilitators to implementing a family-based intervention, and (3) community-level barriers and solutions to optimal heart health to inform the adaptation of SERVE OC’s intervention. Further, this study aimed to examine subthemes for each major theme, including (1) limited CVH knowledge, cultural and gender norms, and misinformation (2) barriers to CVH, including transportation, technology, financial and work constraints; facilitators including CHW and family dynamics (3) community barriers including cost, resources, and environment; community solutions including community infrastructure and access to resources.

**Methods:**

Fourteen focus groups (*n* = 69) were conducted over a 20-month period using a semi-structured interview format. Participants consisted of community family members, community health professionals, and SERVE OC clinical trial participants. Dedoose was utilized to code for thematic analysis, guided by the Social Ecological Model and Social Network Theory.

**Results:**

Three themes were identified: (1) Community CVD knowledge, attitudes, and beliefs; (2) Barriers and facilitators to implementing family-based interventions; and (3) the identification of structural/community level barriers along with community levels strategies to achieving optimal cardiovascular health.

**Discussion:**

Findings showed significant gaps in CVD knowledge and prevention, including understanding of nutrition and barriers to access for healthy foods. Focus groups provided insight into the critical role of familial support in health behaviors and outcomes, and barriers and facilitators for family-based interventions. These results help tailor the SERVE OC family-based intervention in real-time, allowing for a more targeted approach to addressing cardiovascular-related challenges within the Latino community. Through these community-engagement methods, SERVE OC can optimize program design and implementation, maximizing the positive impact of the CVD risk reduction initiative.

**Clinical trial registration:**

http://www.clinicaltrials.gov, NCT05641519.

## 1 Introduction

Cardiovascular disease (CVD) remains the leading cause of death and a major cause of disability worldwide. Disparities in CVD prevalence and outcomes persist within underserved communities, particularly among Latinos in the U.S. with one in four deaths attributed to stroke and CVD (1, 2). Latinos in the U. S. often experience higher prevalence of cardiovascular (CV) risk factors, including diabetes, hypertension, and obesity (3). The Latino community comprises about 34% of the population in Orange County, with about 54.3% of the Latino population in the bottom 20% of self-responding tracts, indicating a disproportionately higher amount of the community facing poverty (4, 5). Latino communities within Orange County are underserved and have been historically discriminated against, leading to numerous health issues and areas of high ethnic population density, which can further exacerbate CVD mortality rates (4, 6). Cardiovascular health (CVH) is influenced not only by individual-level factors but also by broader social processes and environmental factors, such as the social determinants of health (SDOH), which impact health behaviors and contribute to the risk of CVD. While educational health programs are important for CVD prevention, community-driven upstream approaches that address social determinants of health and build social capital are crucial to eliminating health disparities (7). SDOH encompass fundamental factors like education and poverty, as well as proximate factors like limited access to care, neighborhood cohesion, and food insecurity (8, 9). Although access to healthcare and detection of CV risk factors have improved in recent years, efficient strategies optimizing risk factor control have been limited. To effectively prevent CVD within the Latino community, community-engaged research should inform the development of multi-level interventions that address SDOH while targeting individual, community, and policy-level factors (8–12).

For successful community engagement, it is crucial to develop collaboration skills to unite various sectors for designing, executing, and maintaining a successful community intervention that achieves health equity (7). For culturally tailored CVD prevention programs, addressing specific needs and challenges faced by the Latino community is inherent to the success of the initiative (13, 14). Community health workers (CHWs) are essential lay community members that can help reduce disparities by gathering trusted insight on community barriers to CVH, sharing these findings, and advocating for community needs by liaising between the community and researchers (15, 16). For CVD prevention, CHWs can provide culturally tailored educational and information, assist with healthcare navigation, link community members to local resources, disseminate knowledge, and facilitate goal-setting to reduce CVD risk factors (15, 17, 18). Since CHWs are part of the community, they understand the importance of incorporating cultural values, traditions, and preferences into research to stimulate community engagement (16). Collaboration between the community, CHWs, and researchers in developing the study design fosters engagement, inclusivity, and trust between community and researchers (16, 19). Especially for Latino communities, tailoring research to implement a family-centered approach can leverage strong familial ties to promote healthier lifestyles (20). Family-based programs are effective in CV and health promotion, with systematic reviews supporting their role in preventing childhood obesity through culturally adapted, family-driven behavioral changes (21, 22).

Focus groups are another critical component to aid in culturally tailoring the intervention. Focus groups can be used as a pilot strategy to understand barriers communities may face, and integrate those findings into the research design, allowing for improved cultural competency and better adoption of the intervention into the community. Within CVD research, focus groups have been a vital tool to gather data around challenges faced by Latino communities, including access, patient-physician relationships, and understanding of CVD, and to develop more effective tailored interventions (23, 24). Building upon this knowledge, community-engaged research interventions can be tailored to these challenges, such as using community partners to provide better access to nutritional food and safe spaces for physical activity to reduce health disparities within CVH.

Although family interventions and focus group methodologies have been effective, there is little to no research on the implementation of focus groups to understand the interplay between family dynamics and CVH within Latino communities. Focus groups can delve more into the barriers that families face as a unit, and integrate the insight gained from the family dynamic into the research design. Though research has shown various barriers to care like access and safety, there is limited focus group research in CVH has addressed in-depth structural barriers, like transportation or access to resources. As a novel strategy, this research combines both methodologies, and uses focus groups to identify barriers and challenges faced by family units within the Latino community in achieving CVH. A part of the Skills-Based Educational Strategies for the Reduction of Vascular Events in Orange County (SERVE OC), a community-based RCT, this research used focus groups to engage the local community in the research process, and tailor the intervention to address challenges faced by the Latino families.

SERVE OC utilized focus groups prior to the initiation of the trial to enhance, adapt, and culturally tailor the SERVE OC intervention for Latino families and to identify the structural barriers to optimal CVH among the community. As a fluid model, focus group sessions were ongoing during intervention implementation to collect real-time feedback and continuously tailor the intervention to address current community-level barriers and solutions. In this qualitative focus group study, we aim to (1) examine community knowledge, beliefs, and attitudes toward CVD prevention, (2) understand barriers and facilitators to implementing a family-based intervention, and (3) identify community-level barriers and solutions to optimal heart health to inform the adaptation of SERVE OC’s intervention.

## 2 Methods

SERVE OC is a National Institute on Minority Health and Health Disparities (NIMHD) funded trial testing whether a family-based intervention is more efficacious than self-management in preventing hypertension and enhancing CVH as defined by the American Heart Association’s Life’s Essential 8. SERVE OC is adapted from the Discharge Educational Strategies for Reduction of Vascular Events (DESERVE) trial which used a CHW model where CHW’s led educational intervention sessions in a hospital setting to improve self-management and reduce hypertension in stroke survivors (25). DESERVE demonstrated significant results in reducing systolic blood pressure by 9.9 mmHg in the Hispanic intervention group compared to controls (25). As a multidimensional intervention trial, SERVE OC leverages family units to delve deeper into identifying structural barriers to CVH faced by Latino families in the community of Orange County, California. The SERVE OC intervention is focused on improving risk perception, family goal setting, linking families to resources, and intends to foster a supportive family environment that encourages healthy lifestyle choices. The families in the intervention group were given access to the SERVE OC app to track their progress towards their family CVH goals and communicate with their CHWs. Additionally, each family in the study is given a Withings remote blood pressure monitor to allow the researchers to examine trends in blood pressure over time.

### 2.1 Design

This qualitative study conducted fourteen focus groups using a semi-structured interview format over a 20-month period. Four focus group sessions were held prior to the start of the SERVE OC trial, and ten were held throughout the trial. Focus groups only consisted of members of the research team and study participants.

### 2.2 Participants and sampling methods

A total of 69 individuals took part in the focus group discussions. The focus groups included two distinct categories of participants: Community Health Professionals (CHPs) and Latino Community Family Members (CFMs). Of the fourteen focus groups, 14 participants were CHPs, and 55 were CFMs. The sampling method for the focus groups involved a combination of purposive and convenience sampling with the goal of recruiting stakeholders from within community health organizations and from community members. Participants were contacted via email, telephone or spoken to in person to discuss participation in the study and were given an opportunity to ask questions to ensure they understood the aims of the study. CHPs were recruited for the focus groups and were comprised of CHWs and other health professionals (e.g., nurses and other health clinic staff) that were actively involved in provided health-related services to the Latino community in Orange County, California. CHPs were specifically recruited from locations such as the federally qualified health centers and local community organizations in the targeted area to provide unique insight into the healthcare system, surrounding community, and identification of barriers and facilitators to care provision. Conducting focus groups with CHPs was crucial for gaining a deeper understanding of their perspectives, attitudes, and beliefs regarding various health-related concerns, thus supporting intervention development and future programming. CHW-specific focus groups were conducted with community partner organizations including Radiate Consulting and Latino Health Access, recognizing their role as influential peers in promoting positive health actions within communities. All CHPs identified as women, with experience ranging from 1 to 10 years in their respective fields. CFMs entailed family members (including SERVE OC participants) and other community members. These participants were identified as Latino residents of Orange County cities, including Santa Ana, Anaheim, Garden Grove, and Westminster. CFMs were invited to share their experiences and perspectives on their own families and communities.

### 2.3 Theoretical framework

The focus group script and analysis was guided by two theoretical frameworks: the Social Ecological Model (SEM) and Social Network Theory (SNT), which were instrumental in shaping our focus group questions and analysis approach (26–28). The SEM provided a comprehensive framework to explore the multiple levels of influence on CV behaviors and outcomes within the Latino community. This model served as a resource for our focus groups question guide to address factors at various levels, including knowledge and attitudes, interpersonal dynamics, organizational access to healthcare, community characteristics, and broader policy issues.

The SNT complemented the SEM approach by focusing on the role of social relationships in shaping health behaviors and outcomes. This framework guided our exploration of family networks, community connections and social support functions within the context of cardiovascular health. The social support functions, including emotional, instrumental, informational and appraisal support, were integral in understanding social support dynamics within familial relationships.

**Figure 1:**
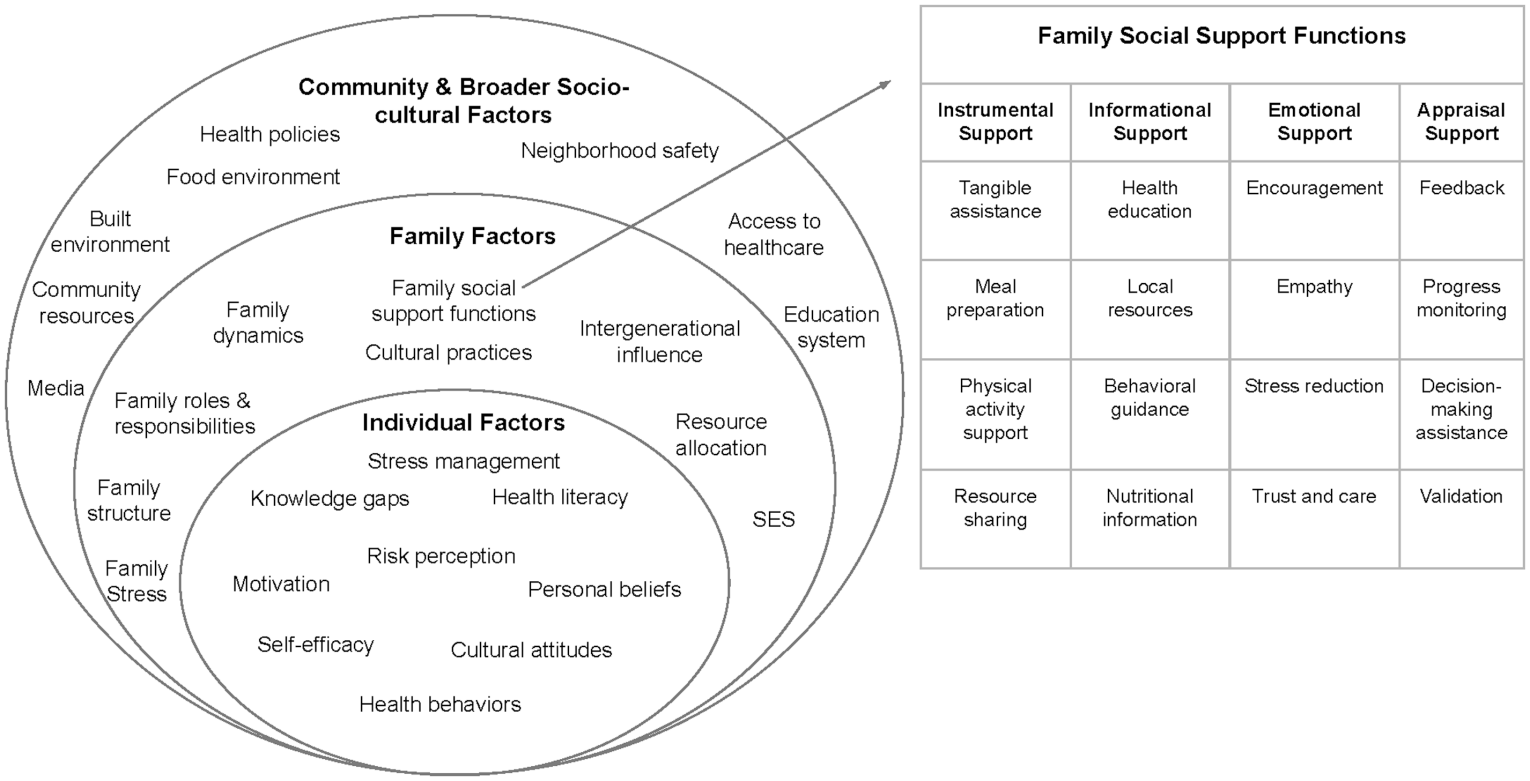
Family-centered Social Ecological Model for cardiovascular health (adapted from the Social Ecological Model to integrate social support functions of the Social Network Theory) (26, 27, 29).

The integration of these theoretical frameworks informed our approach to culturally tailoring interventions, emphasizing the need for a multi-level approach, informing targeting resource allocation, enhancing cultural relevance, promoting social engagement, and fostering community empowerment. This approach aligns with the family-based intervention model employed by SERVE OC, which recognizes that health behaviors and outcomes are deeply influenced by family dynamics, shared environments, and collective habits. Figure 1 illustrates the integration of the SEM with social support functions of the SNT in the context of CVH among Latino families to guide the analysis of focus group data. The figure delineates the various levels of influence in the SEM, with a specific focus on the interpersonal level, elucidating the four key social support functions (emotional, informational, appraisal, and instrumental support) derived from SNT (26–29).

In addition to the SEM and SNT models, community based participatory research (CBPR) strategies were employed, to emphasize the involvement of community members in all aspects of the research process (30). CBPR approaches have shown to enhance the cultural and contextual relevance of interventions, improve relationships between researchers and community members, and facilitate the transition of research findings into practice.

Applying these theoretical frameworks and focusing on family-based interventions within a CBPR approach helped us to develop a comprehensive understanding of the complex factors influencing CVH within the Latino community. This multi-faceted approach was implemented to ensure focus group sessions were culturally tailored and address the multi-faced nature of health disparities within this population.

### 2.4 Research ethics

The focus group study was approved by the University of California, Irvine’s Institutional Review Board under two separate protocols. The first IRB protocol covered the early focus groups prior to the start of the SERVE OC clinical trial, which was approved April 22, 2022 (#1208). The second IRB protocol was created for the SERVE OC clinical trial itself, which incorporates focus groups into the consent form and was approved August 11th, 2022 (#283). Before each focus group, an explanation about the study’s goal and aims was given and informed consent (in which participant’s anonymity and confidentiality was assured) was collected verbally. Additionally, this qualitative study was performed in alignment with the COREQ checklist, which can be viewed in Supplementary Table 1 (31).

### 2.5 Data collection and research personnel

The data collection process for this study was conducted between September 2022 and May 2024, and each focus group interview session lasted approximately 30 to 90 min. Data was collected in various settings, including clinics, churches, parks, and community centers. Focus groups were meticulously scribed in detail by designated bilingual notetakers to document the sessions, with some focus groups being audio recorded when available. The group discussions were moderated by various members of the research team, including the principal investigator (PI), program manager, program coordinator, or graduate student researchers, some of which are listed as authors. Additionally, focus group sessions were assisted by a public health practicum student who took notes in addition to the audio recording. The research team and students conducting the focus groups were both female and male, underwent extensive HIPAA and Collaborative Institutional Training Initiative (CITI) Human Subjects Research trainings, were supervised by the PI, and had previous research experience which equipped each member with the necessary skills to conduct these sessions. To maintain assurance and validity, bilingual team members conducted focus groups either fully in Spanish or English, depending upon the preference and native language of focus groups participants. When bilingual study staff was not available, official translators were hired to provide translation for the discussion. Audio recordings of the focus groups were also later translated and transcribed through the transcription service, Ditto (32), and analyzed to ensure reliability and accuracy of translation by seven study staff members. Data saturation was reached after 14 focus groups, and this decision around saturation was made after the same themes and patterns were observed repeatedly. As no new insights were gleaned from the focus groups, saturation was met as no new themes were added, which stabilized the thematic analysis codebook.

For CFMs who were already enrolled in the SERVE OC study, study staff had already established a relationship prior to the start of focus groups. Other CFMs who were not enrolled in the SERVE OC study did not have a prior established relationship with researchers. CHWs had prior established relationships with the research staff, as CHWs are part of the SERVE OC staff delivering the intervention. Further, the PI has long-standing partnerships with many community stakeholders and is known to the CHWs. Participants were aware the researchers’ reason for conducting this study was to inform the adaptation of the SERVE OC intervention, the study was related to heart health and helping the Orange County community. Positions of the research team were disclosed to participants during focus group sessions. As focus groups were approached as a discussion between research members/group facilitators and participants, transcripts were not presented back to participants for further changes or comments. Additionally, repeat interviews were not carried out. All clarifications on participant answers were discussed in real-time by probing via focus group moderators. Findings were not shared with participants in real-time, however, we continue to share findings with study participants throughout the study, typically at group events. Additionally, a large dissemination event is planned for September 2025.

### 2.6 Materials

The discussion guides for the focus groups were rigorously developed and underwent thorough review by the research team to ensure relevance and clarity. Subsequent real-time revisions were made to enhance their cultural appropriateness, after feedback was obtained from previous focus groups and community partners. A semi-structured approach to questioning was employed during the focus groups to maintain consistency while allowing flexibility to explore emerging topics and encourage active participation. The questions predominantly centered on participants’ personal experiences and perceptions regarding CVD prevention, barriers and facilitators to implementing family-based interventions, and potential community-level barriers and solutions for optimal health. Examples of focus group discussion guide questions can be found in Supplementary Table 2. These questions were reviewed and approved by a layperson within the community and the PI/senior author (B. BA.).

### 2.7 Data analysis

A traditional qualitative approach was taken to capture and analyze data from the focus group sessions. Audio recordings were transcribed verbatim and supplemented with detailed field notes to capture participants’ expressions, and non-verbal cues. Subsequently, the audiotapes, and written notes were analyzed to identify major themes and trends using Dedoose software version 9.2.014 (33).

Themes were informed by the constructs of the SEM and SNT models, which was applied using a deductive approach (26–28, 34). As components of the SERVE OC intervention are being adapted from DESERVE and enhanced with a family-focus for a new setting in Orange County, there were several major themes derived from DESERVE that we were hoping to validate around CVD risk reduction. Therefore, using a deductive approach we utilized the major themes from DESERVE to inform focus group questions and analyses for SERVE OC. For the subthemes, we applied grounded theory and used an inductive approach to further categorize unique reflections from the community and describe the main themes. Hence, themes and subthemes were both identified in advance and after data was collected. Coding of themes was completed by two study staff (D. D. and O. M. H.) separately and compared after to ensure all themes were aligned and agreed upon. A figure of the thematic coding tree can be viewed as Supplementary Figure 1. An iterative process was used to develop coding and work through any discrepancies in interpretation around emerging themes. Discussions helped to reach consensus within the research team. The iterative nature of the analysis process allowed for continuous refinement based on insight gained during successive rounds of topic analysis. Regular reviews and discussions facilitated feedback loops to enhance and identify criteria in response to emerging patterns and themes.

## 3 Results

Following thematic analysis from Dedoose, three overarching themes were generated from the focus groups: (1) Community CVD knowledge, attitudes, and beliefs; (2) Barriers and facilitators to implementing family-based interventions; and (3) the identification of structural/community level barriers along with community level strategies to achieving optimal CVH. Within the first major theme of CVD knowledge, attitudes, and beliefs, subthemes were identified and included limited knowledge of CVD or risk factors, limited knowledge/skills to select healthy options and prepare healthy meals, cultural norms, misconception and misinformation, and gender dynamics. Subthemes for the second major theme of barriers and facilitators to implementing family-based interventions were barriers to implementation (time/work, technology, and transportation), barriers to healthy behaviors, and facilitators to implementation (use of CHWs and familial support). The third major theme, identification of structural/community level barriers along with community level strategies to achieving optimal CVH, encompassed several sub themes including community level barriers (cost, awareness of resources, and environmental spaces) and community level solutions (community infrastructure and school system, and access and resources for nutrition). Table 1 presents these key themes and subthemes identified from the focus group data, supported by demonstrative quotes.

**Table 1:** Quotes exemplifying the themes community CVD knowledge, attitudes and behaviors, barries and facilitators to implementing family-based interventions, and structural barries and community level solutions to achieving CVH and its relevant subthemes.

Main themes	Subthemes	Quotes illustrating themes
Community CVD knowledge, attitudes and behaviors	Limited knowledge of CVD or risk factors	They (community members) cannot recognize the symptoms of heart disease or heart attack, but a lot of… people are feeling… pain in their chest, symptoms like feet swelling. They cannot recognize the symptoms, because they do not understand too much.”
	Limited knowledge/skills to select healthy options and prepare healthy meals	“[An] important thing [to understand] is what are the properties of each fruit or vegetable. Do some have more sugar? Are others more acidic? We do not know a lot of them, so we should know what properties they have. And this would be good [to know] for someone who is diabetic or has high blood pressure to know which foods to eat more or to eat less of.”
	Cultural norms	“Making the change, and that’s culturally what we cannot do, it does not taste good if you do not add lard, right?”
	Misconceptions and misinformation	“I do think we are getting wrong information, but not from health entities like pharmacies or doctors…We are getting misinformation from our families, from our ancestors, our friends. Social media is also where there is a lot of misinformation for the community.”
	Gender dynamics	“[A] Latino guy, [he] feels like a macho man. He…takes some pills and [hopes] he feels better.”
Barriers and facilitators to implementing family-based interventions	Barriers to implementation—time/work	“I do feel that one of the things that we have, one of the factors in our Hispanic community is that we are overworked. We work and we work all the time, and we do not prioritize our health… [Many] do not do a follow up on their health and they only see the doctors in extreme cases when their health is already at risk.”
	Barriers to healthy behaviors	“But I find myself sometimes struggling in getting them (the children) to eat well in a way that is nutritious. But I do not think availability is an issue. It’s a matter of the choices that are easy, that are also available”
	Barriers to implementation—technology	“The technology should be…easy to use. Our community does not have much experience with technology.”
	Barriers to implementation—transportation	“I want to just…go off about transportation…for…the patients and the doctors [transportation is a]…barrier. [I set] up the uber, or the transportation, so that they [can]…attend those…doctor[‘s] appointment[s].”
	Facilitators to implementation—use of CHWs	“… [employing] community health workers from the same area…is a great idea. I think they (the community) will be more receptive to someone who speaks their language, look like them, who has a similar background that will make them understand.”
	Facilitators to implementation—familial support	“We can find support in the family and it is more likely that [we] will follow up after this [CVD intervention] program because [we] can all, as a family, understand the problem, and everyone as a family can follow the [CVD] prevention [program]. So for me, it is very important to have that communication with the community because we know that it is the manner in which we can support ourselves [through family].”
Structural barriers and community level solutions to achieving CVH	Community level barriers—cost	“…make it easier, not so expensive, because if [not]…many of us find it difficult [because]…we are worried about paying the rent, bills, and then focusing on eating healthier, it’s more difficult for us, because…a vegetable already costs you almost 3, 4, 5 dollars per pound, and this is very hard for the community.”
	Community level barriers—awareness of resources	“And I know that there… [are] lots of programs that can help us, but normally when there is a lack of information, lots of people do not know that these programs exist. They exist, but the information does not reach the communities.”
	Community level barriers—environmental spaces	“The situation…[should] not only [rely on] us…[but] on politics…in the people in charge of everything, of us…There are no clean or safe places anymore; we need to put up lights, maintain the little parks to be with our children and the whole family. So, that’s what we have to do, organize ourselves and work as a team to maybe achieve it if it ever happens.”
	Community level solutions—community infrastructure and school system	“When my children were little, there were cooking and nutrition classes. The organizations came and came with their fresh products. And they showed all of us mothers how to make things from healthy pizza with vegetables to salad. It was an hour of class. So, the children, from an early age, from headstart to the first grade, were given those classes to be introduced to vegetables. There are few resources, but this is also a thing I would like to see return.”
	Community level solutions—access and resources for nutrition	“…one of the good things …could be… [building] farms in the communities to grow their vegetables [and] fruits… It would be a good idea … [to] create plots for … each family, and they [take] care of planting.”

### 3.1 Limited community level cardiovascular knowledge, attitudes, and beliefs

The focus group discussions revealed significant gaps in the community’s understanding of CVD and its associated risk factors. Participants highlighted a generalized lack of knowledge around CVD symptoms and recognition, specifically around heart attacks, diabetes, and hypertension. As one CHP expressed, *“We (the community) cannot understand…the symptoms of heart disease or whether a heart attack is happening.”* Limited education and difficulty with understanding medical terminology contributed to the community’s lack of awareness, as one CHP stated, *“They (community members) cannot recognize the symptoms of heart disease or heart attack, but a lot of…people are feeling…pain in their chest, symptoms like feet swelling. They cannot recognize the symptoms, because they do not understand too much.”* This unfamiliarity around CVD extended to the importance of monitoring vascular risk factors, such as blood pressure, and HbA1c, as a CHP noted: *“They (community members) do not know about the conditions or potential barriers [of CV risk factors]. You do not know if they can read or not. It can get very complicated depending on your educational [background].”* CHPs in several focus groups elaborated upon this theme, explaining that many community members were unaware of recognizing critical health behaviors necessary for CVD prevention, such as regular physical activity and healthy eating habits.

Misinformation surrounding CVH was also a prevalent topic of discussion within the focus groups, contributing to the community’s confusion toward CVD. Participants shared cultural myths and inaccurate information circulating on social media or within their families instilled fear and skepticism about CVH and prevention strategies. As one CFM noted, *“I do think we are getting wrong information but not from health entities like pharmacies or doctors but we are getting misinformation from our families, from our ancestors, our friends. Social media is also where there is a lot of misinformation for the community.”* CFMs felt these misconceptions posed significant barriers to their willingness to seek appropriate medical care and adopt preventive measures. A CHP reinforced this point and contributed their perspective to state *“We see many people at the [Federally Qualified Health Centers], but it is usually [when] their health is bad. For example, many Hispanic individuals equate a diagnosis with death… They lack the motivation and are discouraged from seeking [timely] care.”*

Focus groups revealed how deep-rooted cultural norms and other cultural beliefs or traditions shaped the Latino community’s perception of CVD risk factors and hindered the community’s ability to adopt CVH behaviors. One sentiment expressed by many was a sense of a limited understanding of how to prepare nutritious, appealing, and culturally relevant dishes, which compounded the difficulties in transitioning to a diet that supports CVH. Latino CFMs discussed the highly practiced tradition of lard-based cooking and reported adhering to cultural dietary patterns, like eating tortillas, tamales, or sweet breads:

CFM: I eat at least three…tortillas with every meal I eat, with every dinner…

CFM: For me, my family eats a lot of sweet bread with coffee, with coke. So, always with my grandma, who lives in Mexico City … we go in the morning to get bread, go to the bakery. [I say,] “Come on, grandma, let's get some bread in the morning with coffee” … [When it is dinner]. What do we do? We go to the bakery again, and after eating a big dinner, again [we eat more] sweet bread and coffee.

CFM: Another thing, not always, but culturally, we stick to certain things [like] tamales … as a Mexican, the little beans we cooked before with my mom, we cooked them with a lot of lard. So, making the change… that's culturally what we can't do, it doesn't taste good if you don't add lard, right?

However, participants expressed a lack of awareness around the nutritional value of foods (including foods within their cultural dietary pattern), reading nutrition labels, appropriate portion sizes, and the impact of key dietary components like sugar, salt, and calories. This challenged CFMs to make informed choices about the foods and quantity of foods they consume:

CFM: … another important thing [to understand] is what are the properties of each fruit or vegetable. Do some have more sugar? Are others more acidic? We don’t know a lot of them, so we should know what properties they have. And this would be good … for someone who is diabetic or has high blood pressure to know which foods to eat more or to eat less of.

CFM: In thinking about the fresh vegetables, sometimes you don’t know how to prepare them. We need to know how to prepare them because, a lot of the time, you have to throw them away since you aren’t familiar. We need them to help us know how to prepare those vegetables. Because it is a lot, but what do we do if [we] aren’t familiar with them? We need to be familiar with them.

CFM: …I find that I don't know well enough [about counting calories] […That's a big problem because… I think the main components about healthy eating [are] also [about] caloric content…

CFM: And the quantities we [eat]. Of course, when we had no food, it was different. But the feeling now, that we have food, we fill the plate with a lot of food, so … when you eat everything and don't combine it with options, vegetables, etc., culturally, that's what happens.

Cultural norms, attitudes, and beliefs around nutrition became a central point of focus within the focus groups. Many CFMs within the focus groups struggled to identify healthy meal options beyond the example of a simple salad. The lack of nutritional awareness around heart-healthy meals and cultural dietary patterns significantly contributed to the community’s challenges in achieving CVH.

Aside from nutrition, focus groups shared how cultural customs influenced family dynamics and the traditional gender norms within the Latino community, which created obstacles to CVH. Focus groups gave insight into the machismo culture, highlighting a shared male view of their expectation to prioritize work and avoid seeking medical attention, unless prompted by their spouses. Male CFMs provided further commentary:

CFM: … I refuse to go to the doctor. But my wife push [es] me all the time…

CFM: … a Latino guy feels like a macho man. He thinks like oh I have a headache… [he] take [s] some pills and hope [s] he feels better.

Families shared how this power dynamic makes it challenging for women to encourage their male partners to adopt heart-healthy habits or seek medical attention, despite their recognized role as the primary caregivers within the family.

### 3.2 Barriers and facilitators to implementing family-based interventions

#### 3.2.1 Barriers to implementation

Participants highlighted several practical barriers that could hinder their ability to engage in family-based health programs. Transportation and work schedule challenges were primary concerns, especially pertaining to access of healthcare services and CVH. Many participants cited a lack of reliable transportation and inconsistent public transit were obstacles they faced in reaching medical appointments, since it limited their options and often conflicted with their work schedule. As one CHP stated, *“I want to just … go off about transportation … for …the patients and the doctors [transportation is a] … barrier. [I set] up the uber, or the transportation, so that they (the patients) [can] … attend those… doctor[‘s] appointment [s].”* The demands of multiple jobs or precarious employment significantly compounded the effects of time and transportation issues for many CFMs. Within the Latino community, participants described a cultural emphasis of being overworked that often prioritized employment over health to make ends meet, as a CFM stated, *“I do feel that one of the things that we have, one of the factors in our Hispanic community is that we are overworked. We work and we work all the time, and we do not prioritize our health … [Many] do not do a follow up on their health and they only see the doctors in extreme cases when their health is already at risk.”* Focus group participants underscored how these stressful work schedules created barriers to CVH and could further inhibit the SERVE OC intervention as a CHP commented, *“I think a lot of that (participation and follow-up) might be difficult if they are working late or work two jobs.”*

Due to work, time, and financial constraints, participants felt like they had to sacrifice nutrition, exercise, medical appointments, and other healthy lifestyle choices. As one CHP noted, *“When we are dealing with prevention, we may go to a doctor and the doctor may say, take this medication, follow this diet, and the diet may say eat fruits and vegetables. However, because we run from here to there, from there to here, we may have two jobs. We may not have enough time or finances to adhere to the diet that goes with the medication.”* CFMs articulated the obstacles they face in obtaining affordable, healthy food due to their socioeconomic circumstances, as one CFM shared, *“…healthy foods, vegetables, all those things would help a lot … if they were a little more accessible to the customer because…if you look at the prices, many people get scared [at] places like Whole Foods that have fresh vegetables [that] are more expensive and… inaccessible for many people.””* For families, nutrition was the first to go when facing pressure with time and work schedules, as a younger CFM stated*, “I ate a lot of fast food, [for example] McDonald’s or chicken nuggets. They (parents) are very busy with work, and when [they] come back from work, they are tired.”* Focus groups revealed that parents struggled to find time and try different recipes to prepare healthy meals. Some participants suggested the importance of teaching their kids how to cook, so children can grow up learning how to make healthy meals instead of relying on parents inflicted by time constraints. One CFM shared, *“So, they (children) should be there in the kitchen when the parent is cooking because, as you say, they are very busy with work, and when they come back from work, they are tired. There’s no time. And that’s when we go out to buy, and that’s not very good.”* CFMs further elaborated to explain they often turned to buying pre-pared or fast food since they felt limited in their choices for convenient, healthy foods that also appealed to children. As one parental CFM shared, *“But I find myself sometimes strugg [ling] in getting them (the children) to eat well in a way that is nutritious. But I do not think availability is an issue. It’s a matter of the choices that are easy that are also available.”* For families with children that have very selective palettes, they felt even more limited in their options. As one younger CFM shared, *“Well, I was a very picky child. I did not eat many good things. And my mom had a hard time finding food that I would eat. But for a long time, we were trying to make the food comfortable for me. And I ate a lot of fast food, McDonald’s or chicken nuggets … I’m very picky.”* Focus groups showed that participants often prioritized convenience over health, opting for less nutritious food options due to a limited availability of healthy, tasty, and readily accessible meals. Many participants were aware that this dietary shift negatively impacted their CVH.

Identifying this barrier, the SERVE OC intervention was tailored to provide an abundance of resources for study participants, including quick, healthy recipes, via the SERVE OC app. When addressed in the focus groups prior to the start of the trial, CHPs found the lack of access to technology and the digital divide was a barrier that could limit the reach and engagement of family-based interventions. Participants expressed concerns about their ability to access online resources or lack of understanding of how to use technology. CHPs further commented on the technological barrier:

CHP: The technology should be something that is easy to use. Our community does not have much experience with technology.

CHP: We have to remember that this application (SERVE OC app) is going to be used by the parents … grandparents, or by people who were not born with that technology.

#### 3.2.2 Facilitators to implementation

Focus group findings emphasized the critical role of social support from family and the community as key facilitators for successful implementation of family-based CVD prevention programs. Discussions touched upon examples of informational, instrumental, emotional, and appraisal assistance from their family support network in helping them adopt and maintain heart-healthy behaviors. As one CFM noted, *“… We care a lot about family–so connecting with families or making that connection [makes it so much easier to take care of my health].”* Informational support encompassed the education and familiarization of families as a whole to help reduce the risk of CVD. CHPs emphasized the importance of educating families to reduce the fear of seeking medical attention and capitalizing on the family-based SERVE OC intervention to promote CVH:

CHP: We need to educate them (family members) to not be afraid and go to the doctor and tell the doctor how they feel [or another family member feels,] so they can monitor them and find out if it is high or low blood pressure.

CHP: I think these organizations should work with the entire family. I think it will work. They maybe call out for workshops maybe once a month and during the workshop they can have a basket full of healthy products that will be given to the families that come. They also can incorporate exercise as the families come to the workshop. This could be an ongoing monthly thing where they can also …giv [e] [out] blood pressure monitors. They can be taught how to use them. They can even …giv [e] agendas where to write this down. There are so many resources where they can teach the importance of following up with these topics.

The concepts of instrumental and social support also emerged around discussions that families provide tangible assistance to help with the blood pressure monitoring device. Prior to the trial start, CHPs discussed how familial support could be leveraged to help the older generations take their blood pressure. One CHP shared *“…the patients check their blood pressure at home. And how about [the] BP monitor?… the blood pressure is a little bit different from [when] people get here. And in that case, we do involve the family. [We ask], ‘How are you? Are you checking your blood pressure? What medications are you on?’ Especially the older population, they tend to be [un] motivated or they have pretty bad vision. And so, the [older] children will take over their medication management. And that’s whom we ask about [support with] adherence. So that’s how we are able to know [how helpful the family member is.].”* During the trial, findings from the focus groups showed families work as a unit to setup the equipment, help with proper cuff placement, record measurements, and transmit data to healthcare providers. One CFM expressed, *“In our family, we always help each other out. Learning to use the blood pressure device together wasn’t just about health; it was about showing that we care and that we are in this together.”* Participants felt empowered by this instilled familial support in the intervention. Instrumental support extended beyond operation of the device, as many of the older generations within families shared how their supportive family network reminded them to take measurements regularly. Focus groups also highlighted appraisal support in CVD risk reduction, as family members provide feedback and encouragement, monitor progress, assist in decision-making, and validate efforts toward healthy lifestyle changes. One CHP suggested, *“They can be taught how to use them (blood pressure monitors). They can even be giv [en] agendas where to write this down. There are so many resources where they can teach the importance of following up with these topics.”*

The focus groups confirmed that by leveraging these familial and community support networks, participants could overcome practical barriers and sustain their engagement in CVD prevention efforts. Emotional support from the family network was seen as a crucial factor. As one CFM expressed, *“We can find support in the family and it is more likely that [we] will follow up after this [CVD intervention] program because [we] can all, as a family, understand the problem, and everyone as a family can follow the [CVD] prevention [program].”* Overall, CHPs and CFMs agreed upon the importance of a family support system in preventing CVD.

Another critical avenue of support for implementation of the SERVE OC intervention outside of the family network was through CHWs. Participants expressed a high level of trust and connection with CHWs, who were seen as integral members of the community. As one CHP shared, *“… [employing] community health worker from the same area … is a great idea. I think they (the community) will be more receptive to someone who speaks their language, look like them, who has a similar background that will make them understand.”* Participants expressed that having access to culturally congruent CHWs who provide education, guidance and feedback empowers the family unit and community to make informed decisions, sustain their engagement, and commit to lifestyle changes that promote CVH. As one CFM expressed, *“So for me, it is very important to have that communication with the community because we know that it is the manner in which we can support ourselves [through family]. Also the community will feel heard because it is people like ourselves [community health workers] teaching, and therefore will pay more attention to the information.”* Through the SERVE OC intervention, CHWs were key administrators in supporting participants by addressing challenges with time and available resources. This included offering flexible schedules with study events to accommodate participants’ work commitments and building strong relationships through in-person interactions to promote CVD prevention resources. CHPs were vital in planning the intervention and giving a roadmap for CHW community engagement, as several CHPs gave their suggestions on ways to mitigate these barriers:

CHP: Perhaps that is something that we can do: Go out door to door to give out a flier that directs people to information about hypertension while inviting them to the park to participate in physical activity on weekends. Weekends tend to be the days where people have the time to participate. That way we can involve them in education and physical activity. At the same time we can have an area dedicated to kids.

CHP: Because we, that are community workers, have noted that the Latino community is more likely to receive [internalize] information when it is given to them personally and when we [community health workers] are present with them and explain what prevention is and what it involves. In this case where it [information] will be given to families, it is better. Because it is a class on health that will show families what the problem is and what they need to do as a group.

### 3.3 Structural barriers and community-level solutions to achieving CVH

#### 3.3.1 Community-level and structural barriers to achieving CVH

Accessing and affording healthcare services was a key community level barrier to enhancing CVH. This challenge was multifaceted, encompassing issues of cost, insurance coverage and the barriers created by financial concerns. As one CHP explained, *“… I have found that there is a lot of fear of people going to the clinics because of how expensive they may be or the lack of medical insurance.”* Focus groups elaborated on the lack of access to healthcare, and the lack of promotion around available resources, as one CHP noted, *“The problem is a lot of people do not have the insurance or the information. They do not know about these programs. They have limited knowledge as to where to go. They do not have the information.”* Identifying this barrier helped to explain participants fear around CVD, and placed importance upon SERVE OC to work with the community in finding strategies for improving healthcare access and advocacy around resources while emphasizing the importance of adopting behavioral health changes as a preventative measure.

Participants also expressed concerns about their surrounding food environment, as families noted a limited availability of affordable, quick, healthy food options within their community. A CFM shared*, “I lived in an area [where] the closest grocery store is more expensive than if I traveled further… if I go to Northgate, their vegetables are so much cheaper and their fruit, …”* Participants stressed that healthy options are a luxury, and defaulted to fast-food because it is cheaper, cost-effective, and readily available. Many people in the community have other financial priorities that take precedence over healthy eating, as participants expressed their concerns:

CHP: … make it easier, not so expensive, because if [not] … many of us find it difficult [because] … we are worried about paying the rent, bills, and then focusing on eating healthier, it’s more difficult for us, because… a vegetable already costs you almost 3, 4, 5 dollars per pound, and this is very hard for the community.

CFM: … spend [ing]… money at whole foods or trader joes, which sometimes does not have such good (affordable) prices… For a meal for example, a lettuce… may be cheaper elsewhere than at Whole Foods. It is also sometimes more convenient to go to McDonalds, [where some meals] are one dollar.

CFM: … healthy foods, vegetables, all those things would help a lot if they were a little more accessible to the customer – because…[at] Whole Foods … if you look at the prices, many people get scared

CFM: Now, with the economy, everything is too expensive.

Focus group participants also discussed a lack of community-based programs, tangible aid, and other resources that could provide education or support around CVD prevention. While some participants felt there were not enough resources, others emphasized there were a lot of resources, but were inaccessible due to a lack of promotion and communication to community members. One CHP noted, *“And I know that there… [are] lots of programs that can help us, but normally when there is a lack of information, lots of people do not know that these programs exist. They exist, but the information does not reach the communities.”* The discussion delved deeper and focused on the barriers of health literacy and language concordance as two blockers to access of resources. Participants shared the same sentiment that even when CVH information is presented, it is riddled with scientific or medical jargon or is not in their primary language, which prevented them from understanding the CVH resources and worsened their disengagement. Regarding health literacy, parents and older generations within families recognized their lack in knowledge around CVD, and emphasized a need for change so the younger generations are more prepared in addressing risk factors, healthy behaviors, and chronic disease management. One CFM expressed, *“I think that teaching them (my kids) this information about [how] you can grow this food in your own backyard [is helpful] … before my own tragedies [my kids] were not on the right trajectory. They were eating terrible food, whatever was fast … And now we have to take the time to actually prepare it and make it [healthy] and get that [good] influence in them … “*Many parents within the focus groups highlighted a barrier to CVH education, and the need for change at the community-level through school education and nutrition for their children. One CFM shared*, “I think it starts in the beginning … with the kids [getting started] as early as possible…[on] any type of [CVD prevention] intervention where they are learning [about healthy eating and nutrition]…”*.

Participants also highlighted the lack of safe, accessible, and supportive community environments that could facilitate physical activity and healthy lifestyles. Concerns were raised about the absence of large green spaces, safety of parks, and well-lit areas for exercise. One CFM stated, *“There are spaces, small parks, but they are full of gangs and people smoking, and many things. So, the spaces where we live are not safe; you do not feel safe to go out for a walk.”* Another CFM expressed, *“… we do not have places or spaces to walk, to exercise.”* Families noted that exercising together through sports like soccer or going on walks was important in bonding and achieving CVH, but the state of the surrounding neighborhood environment inhibited them from these activities.

#### 3.3.2 Community-level solutions to achieving CVH

Participants suggested a range of community-level solutions to address identified structural barriers. Significant focus was around increasing access to affordable, community-based programs and resources that provide education, skills training, and support for adopting heart-healthy behaviors. Families emphasized the importance of incorporating CVH education and skill-building into school curricula. To address the generational difference in health literacy, many CFMs discussed how families turned to the schools and local organizations to incorporate CVH in education and nutrition:

CFM: For my children, I have asked that they make changes in the schools in the Anaheim district.

CFM: Their (children) school they replaced everything. They've amped the entire menu. They have to have so many vegetables and fruits [o] n their tray with their food, with the milk. So it's [now] a full complete [healthy] meal.

CFM: Our school does a once-a-month food thing where there's fruits and vegetables available. Also, she (my child) can go in and just get the fruit. She could go in and… get the healthy things … that is available and an option. This should be how it’s done in other schools.

CFM: When my children were little, there were cooking and nutrition classes. The organizations came and came with their fresh products. And they showed all of us mothers how to make things from healthy pizza with vegetables to salad. It was an hour of class. So the children, from an early age, from headstart to the first grade, were given those classes to be introduced to vegetables. There are few resources, but this is also a thing I would like to see return, the nutrition classes in schools …

Although steps were taken to initiate community-level changes by asking schools for healthier lunch options or teaching knowledge around health, families expressed a main motivation for taking part in the SERVE OC intervention was to help teach their children about CVH and surround themselves with a supportive community in perpetuating healthy changes.

Alongside nutrition, many participants were passionate about the community’s role in addressing barriers to accessible healthy food options and the nature of healthy eating. Many participants suggested that local government should provide access to free or low-cost healthy foods at food banks, community centers, or farmer markets as a critical solution. Several CFMs shared their views:

CFM: … we would like for there to be more markets like this in which the foods are organic–they don’t have chemicals or pesticides.

CFM: … access to affordable, healthy food is tough around here. If we had more community help or assistance, it would really make it easier for families to make healthier choices.

Participants underscored the importance of the community coming together to instill community gardens and urban farming initiatives that empower families to grow their own produce and gain greater control over their food sources. As one CFM explained, *“… one of the good things … could be… [building] farms in the communities to grow their vegetables [and] fruits… It would be a good idea … [to] create plots for … each family, and they [take] care of planting.”* Implementing these solutions to make nutritious options more affordable and accessible, the next step is promoting resources for recipes and ideas that support CV wellness. CHPs discussed the role of community in providing accessible programs, like cooking classes, to promote awareness of healthy foods:

CHP: … I think even recipes, even like doing a couple cooking classes for different types of … recipes would help someone to plan rather than having salad every night.

CHP: I feel that these workshops helped families a lot in understanding the idea of balanced healthy diets and the prevention of illness.

The majority of the focus groups discussed the lack of available options, and lack of awareness around community organizations and governmental agencies that provide resources on CVH and nutrition. Focus groups emphasized the need for increased community outreach and awareness-building efforts, utilizing various channels (i.e., newspaper, social media, flyers, door-to-door interactions) to promote CVH. One CFM shared, *“I think that for us to find out about the organizations and eat more healthily, they should put up more flyers …”* Participants believed that widespread dissemination of information about CVH (including resources), risk factors, and prevention strategies could help mobilize the community and foster behavioral change.

Focus groups emphasized the importance of using the SERVE OC intervention as a building block and platform to involve the surrounding community to address barriers to communication and lack of awareness of healthcare resources. Discussions underscored the value of CHWs and community-based organizations in providing outreach, education, and culturally congruent programming. As one CFM expressed, *“I … appreciate when they explain things in a way that’s easy to understand. Knowing what activities are best for our family and getting [practical] tips and recipes from each other helps us make better choices [together]”* Participants emphasized the importance of transforming the findings from the family-based intervention into community-level solutions, and unifying not only as families, but as communities to support one another in achieving CVH.

Aside from improving outreach, participants identified a lack of safe neighborhood and built environments as a significant obstacle to CVH. Focus groups advocated for increased involvement from local government and policymakers in developing community spaces that encourage physical activity and family-oriented wellness programs. As one CFM mentioned, *“The situation … [should] not only [rely on] us … [but] on politics … in the people in charge of everything, of us … There are no clean or safe places anymore; we need to put up lights, maintain the little parks to be with our children and the whole family. So, that’s what we have to do, organize ourselves and work as a team to maybe achieve it if it ever happens.”* Focus groups unanimously agreed upon the importance of coming together as a community to protect outdoor spaces, and address issues of safety and security. It was important for families to have safe spaces outdoors to exercise and bond together, as one CFM noted, *“…they are creating a skateboard park, and kids love that. These are things that we lack, having those spaces, and also spaces like this (parks) where we can share our stories and learn what works for [us as] a family.”* Suggestions included improving available green spaces to become cleaner and more secure, access to more parks, and more well-lit areas for physical activity that unifies the community in achieving CVH.

## 4 Discussion

This qualitative study provides important insight into community specific knowledge, attitudes, beliefs, barriers, and facilitators toward CVD health and prevention in Latino families residing in Orange County, California. Overall, the study elicited three core themes throughout all focus groups: (1) Community CVD knowledge, attitudes, and beliefs; (2) Barriers and facilitators to implementing family-based interventions; and (3) Structural barriers and community level solutions to achieving CVH. These themes encompass specific focus group findings regarding community knowledge gaps, families and social support, influence of cultural norms on healthy behaviors, and practical and social factors affecting intervention implementation. Results also highlighted challenges related to nutrition and food security, significant structural impediments and potential community-driven solutions. Additionally, the study sought to explore these structural-level challenges and community-driven solutions to achieving optimal CVH, in order to inform the development and implementation of a culturally tailored, family-based CVD prevention intervention by SERVE OC. Specifically, these findings tailored the SERVE OC multi-level intervention approach through assessing the appropriateness of a CHW-led intervention, enthusiasm for a family approach, and utilization of community-engaged discussions with governmental and non-governmental organizations (NGOs) around structural level barriers.

The focus group findings revealed significant gaps in the community’s knowledge, attitudes, and beliefs regarding CVD and its prevention. Participants demonstrated a lack of understanding about the symptoms, and risk factors, which extended to the importance of monitoring vital signs, such as blood pressure, and recognizing early indicators of potential CVD. Focus groups highlighted the role of misconceptions and misinformation, thought to be perpetuated through cultural myths, social media, and within families. Indeed, deeply rooted cultural norms and gender dynamics also posed barriers to optimizing CVH behaviors as men were often reluctant to prioritize their own well-being or seek medical attention. These findings align with literature reporting the negative effect of a cultural dynamic where some Latino men need to appear strong and self-reliant, which discourages them from proactively managing their health and engaging in preventive care (35). Further, the findings suggest that the Machismo culture can also impact women’s ability to influence their partners’ health behaviors (35, 36). Focus groups also reported on issues around access to prevention services. Inaccessible hours, cost, or location make it challenging for workers to prioritize their health, as they may struggle to find time for preventative care or healthy lifestyle choices. This avoidance and culture of being overworked can result in undiagnosed or poorly managed conditions such as hypertension or diabetes, all of which are significant risk factors of CVD (37). The demands of precarious work can also limit individuals’ ability to engage in health-promoting activities, such as regular exercise or healthy meal preparation. Moreover, the insecurity associated with precarious employment can lead to reliance on unhealthy food options and delayed medical care, further compounding health risks (38, 39). SERVE OC incorporated this feedback into the intervention by altering hours for appointments to align with precarious work schedules, like scheduling events and follow-ups over the weekend. These findings underscore the importance of SERVE OC’s family-based intervention, which leverages familial bonds to unite families and foster mutual support. SERVE OC intervention activities are further tailored to involve family-based events that challenged gender dynamics and fostered inclusivity for male participation in activities by engaging families as a unit to prioritize their health, like walking groups or urban farming events. Thus, focus groups supported family-based interventions as an effective method to reach all members within the family to better CVH.

Additionally, the findings highlighted the multidimensional complexities surrounding nutrition and CVH, as all three themes extracted from the focus groups mentioned the impact of nutrition. Results from the focus groups uncovered significant challenges in adopting heart-healthy dietary practices due to traditional cooking methods, preferences for convenience foods, and a lack of nutrition knowledge and literacy around meal preparation, portion sizes, and food nutritional value. A more nuanced comment around families struggling with the transition from food scarcity to food abundance captures the complexity of sustained nutritionally healthy behaviors. A lack of nutrition literacy compounded the difficulties in transitioning to a diet that supports CVH. Moreso, dimensions to the problems expand further to encompass the lack of access to healthy foods, growing cost of healthy foods, and lack of time to prepare healthy meals, further inhibiting CVD prevention techniques and strategies to be sustainably adopted. There is a growing body of literature slowly beginning to untangle the multidimensional challenges around healthy food behaviors and enhanced CVH, which stress the influence of social, cultural, and environmental determinants of health (40, 41). Cultural norms around traditional cooking practices, like using lard-based cooking, can significantly detract from attaining optimal CVH. Social and environmental determinants such as health literacy around nutrition, cost, lack of access, and food environment also hinder achieving CVH, which can lead to the increased uptake of ultra-processed convenience foods (40, 42). Research shows that diet quality is poor within the US, and the disparity gap in nutrition has increased between socioeconomic classes over the years, mirroring the increasing health disparities gap in CVD in vulnerable, disenfranchised communities (40–42). Poor dietary nutrition can lead to chronic inflammation, reduction of diversity within gut microbiome, hypertension, diabetes, and more, further contributing to the development of CVD (40). More work partnering with community and community-engaged research methods is needed to overcome the significant barriers to robust nutritional lifestyles in diverse communities (40). This study highlights the immediate need to focus on not only education around good nutrition, but on taking a systems-level approach to healthy nutritional behavior and partnering with communities. One recent community-based participatory research study showed the efficacy of using a culturally tailored, multilevel, church-based intervention in significantly reducing BMI and sustained healthier dietary changes in African American and Latino communities in Los Angeles (43). Regarding improving health literacy in vulnerable populations, a statement by the American Heart Association recommended several key strategies, including a universal toolkit, health mobile apps, patient narrative videos, and CHWs (44). These strategies have been implemented into the SERVE OC intervention to assess their impact on improving health literacy. Due to the focus group findings, our intervention for SERVE OC was tailored to include more focus on nutrition, including culturally adapted cooking classes, multigenerational meal planning, and working with local food providers and organizations within the communities. Thus, partnering with local communities by involving local healthcare professionals, public health initiatives, and community-based interventions such as SERVE OC to improve access to healthy foods, increase health literacy, and understanding around nutrition may be a sustainable path forward to improving CVH and CVD prevention.

The focus group findings also underscored the critical role of family networks and community-based social support in facilitating behavior change and sustained engagement in CVD prevention efforts. Participants emphasized the importance of different aspects (emotional, instrumental, informational, and appraisal) support from their loved ones and trusted community members. A number of studies have described the potential impactful role of families in lowering vascular risk (45–49). The focus group discussions revealed a nuanced understanding of the barriers and facilitators that could impact the implementation of family-based CVD prevention interventions. Participants highlighted practical barriers, such as transportation challenges, competing family responsibilities, and lack of access to technology, that could hinder their ability to engage in health-promoting programs. However, the findings also underscored the critical role of social support as a key facilitator for successful implementation, for example, the importance of family members in bridging the technological gap, especially for older adults or those less familiar with digital devices. Additionally, family members can provide support with related tasks that indirectly impact blood pressure, such as assistance with medication adherence, preparing heart-healthy meals, or facilitating physical activity. The importance of this type of support is further emphasized in the context of cultural values that prioritize family involvement in healthy matters, particularly Latino communities (50, 51). By leveraging these multifaceted forms of social support and addressing the practical barriers faced by the community, the findings from this study can help SERVE OC develop more effective and sustainable family-based interventions to improve CVH outcomes within the community. For example, SERVE OC community-based cooking classes can teach all generations about healthy nutrition while participating as a family unit. SERVE OC also encouraged younger generations to support older family members with technology, such as the SERVE OC app and at-home blood pressure monitors, which fosters intergenerational support and helps close the digital divide. In addition, leveraging those family networks and providing families with the necessary knowledge and tools, healthcare interventions can enhance the effectiveness of home blood pressure monitoring programs and improve overall CVD prevention.

Beyond the individual and interpersonal factors, the focus group discussions revealed key structural barriers that impede optimal CVH within the community. Participants highlighted challenges related to limited healthcare access and lack of healthy food availability. In response, the community proposed a range of solutions, including increased access to affordable educational programs and the establishment of community gardens. Specifically, the community elaborated more on structural barriers that contribute to unsafe community environments, discouraging physical activity. Community members generally agreed that the safety of outdoor environments is influenced by neighborhood safety, homelessness, and immigration-related fears. Under-resourced communities within Orange County have higher densities of homelessness, including Santa Ana and Anaheim, contributing to homeless encampments at parks and outdoor spaces which can create safety concerns (52–54). Certain communities in Orange County have faced historical disinvestment and redlining, which can result in higher rates of community violence (55, 56). Neighborhoods with a history of community violence can lead to community members feeling unsafe to exercise outdoors, limiting their opportunities for physical activity. These insights highlight critical issues that can inform collaborative efforts between the community and local government to improve CVH, like developing parks in safe areas, increasing security around established parks, strengthening policies that support and protect immigrant communities in alignment with California’s sanctuary law, and improving outreach efforts to reduce homelessness.

These findings validate other data around the importance of advocacy in community-engaged research and inclusion of community members within the study design and intervention (57–59). Similar to our study, one mixed-methods study used focus groups and surveys to analyze the efficacy of their Community Advisory Board, which highlighted the importance of key stakeholder involvement in community-engaged translational research due to their diverse background, inherent ties to the community, and ability to effect change (60). A recent statement by the American Heart Association emphasizes the need for community partners and key stakeholder involvement in community-engaged strategies to improve CVH within local communities (61). This study supports current literature on the necessity of community-engagement to increase resources and access to healthcare by involving community partners, stakeholders, and local government. To address structural-level barriers, SERVE OC is taking steps to improve CVH in Latino families by involving community partners and the Community Advisory Board to find best methods to disseminate the findings of the SERVE OC clinical trial back to the community and working together to determine next steps. This can include working together with local government to enact policies that will improve CVH outcomes. Future research should further explore the effectiveness of community and family-based interventions that address individual and structural-level barriers to improve CVH outcomes in Latino families.

## 5 Limitations

While this study provides valuable insight into the CVH knowledge, attitudes, and behaviors within the target Latino community, as well as the barriers and facilitators to implementing family-based interventions, there are several limitations that should be acknowledged. One limitation is the reliance on self-reported data from the focus group participants, which may be subject to social desirability bias and not fully reflect the participants’ true experiences. To limit this, multiple focus groups were conducted and study staff was trained to guide the conversations when needed, but mainly help participants themselves lead the conversation. Another limitation was the variability in translation and transcription in focus groups since multiple bilingual staff and translators were used, which could lead to incomplete themes or quotes from focus groups. Mitigation was attempted by using a software for translation (Ditto) and multiple bilingual study staff or translators collaborating together on translations. Additionally, the focus group methodology, while providing rich qualitative insight, has inherent limitations in terms of sample size and generalizability, as the perspectives shared may not be fully representative of the broader Latino community within the region. Expanding the research to include a larger, more diverse sample through surveys or individual interviews could help validate and further contextualize the findings.

## 6 Conclusion

This research study aimed to gain a comprehensive understanding of the factors influencing CVH within a target Latino community, in order to inform the development and implementation of a culturally tailored, family-based disease prevention intervention by SERVE OC. The utilization of focus groups combined with a family-based intervention underscores SERVE OC’s commitment to community-driven insight through implementing a novel strategy that aims to enhance the overall effectiveness of the CV risk reduction initiative. The focus group discussions revealed significant gaps in the community’s knowledge about CVD, its risk factors, and prevention strategies. This community-engaged, participatory approach is crucial, as it allows researchers to deeply understand the community’s perspectives and collaboratively design solutions that are culturally responsive and sustainable. Future research should expand upon these qualitative insights by conducting quantitative studies that validate the prevalence of identified barriers and facilitators in larger Latino populations, and evaluate the effectiveness of family-based interventions like SERVE OC in improving CVH outcomes.

## Data Availability

The datasets presented in this article are not readily available because in the IRB protocol (#1208) for early focus groups we stated we would not share our data. For our later focus groups under IRB protocol (#283), we are only allowed to share de-identified data upon request. Requests to access the datasets should be directed to Bernadette Boden-Albala, bbodenal@hs.uci.edu.

## Supplementary material

SUPPLEMENTARY TABLE 1 Completed COREQ (Consolidated criteria for reporting qualitative research) Checklist. This checklist is developed from Tong et al. (31).
