# Supplementary material for: Knowledge, barriers, and facilitators for promoting cardiovascular health in a Latino community: a qualitative sub-study of the Skills-based Educational Strategies for Reduction of Vascular Events in Orange County
Source: Front Public Health. 2025 Jun 18;13:1531775. doi: 10.3389/fpubh.2025.1531775 (PMC12213769; doi:10.3389/fpubh.2025.1531775)
Supplement: Supplementary file 2 [file Table_2.DOCX]

**Supplementary Table 2**. Example Questions From Focus Group Discussion Guides

| 1. When you think about your own health, and the health of your family and community, what are the current needs for promoting/adopting healthier diets (more fruits, vegetables, water, whole grains, good fats, lean meats/poultry/fish, less salt, bad fats, added sugar, and fast food)? 2. What are some strategies that you and your family can use to ensure you work together as a family unit to be more physically active, eat healthier, and monitor your blood pressure together? 3. What activities do we enjoy doing together as a family that help us relax and have fun? 4. What are some resources that would be helpful for learning about healthy eating and exercise and being physically active to promote heart health? 5. How has the COVID-19 pandemic affected the accessibility and availability of healthy food options for you, your family, and your community? 6. What might be some barriers or challenging factors when we deliver or implement this intervention? 7. Although local food programs serve as a critical resource for families to alleviate some financial strain, how do you feel about the nutritional value you may be receiving? 8. Can we think of one new activity we'd like to try together as a family to relieve stress, and how can we make it happen? 9. What are some resources that would be helpful for learning about healthy eating and exercise and being physically active to promote heart health? 10. What are the most effective communication strategies for residents to learn about these events and resources? 11. How do residents learn about programs and events to promote physical activity and healthy eating? 12. What are some strategies that are helpful for ensuring that residents are able to continue to use the tools and skills from the program after it ends, such as being physically active, healthy eating, and monitoring their blood pressure as a family unit? 13. What are factors or successful strategies that support physical activity among Latino residents? 14. What are factors or successful strategies that support healthy eating among Latino residents? 15. What have you experienced or seen that has been effective when working with children? |
| --- |
