# Supplementary figures and images for: Knowledge, barriers, and facilitators for promoting cardiovascular health in a Latino community: a qualitative sub-study of the Skills-based Educational Strategies for Reduction of Vascular Events in Orange County
Source: Front Public Health. 2025 Jun 18;13:1531775. doi: 10.3389/fpubh.2025.1531775 (PMC12213769; doi:10.3389/fpubh.2025.1531775)

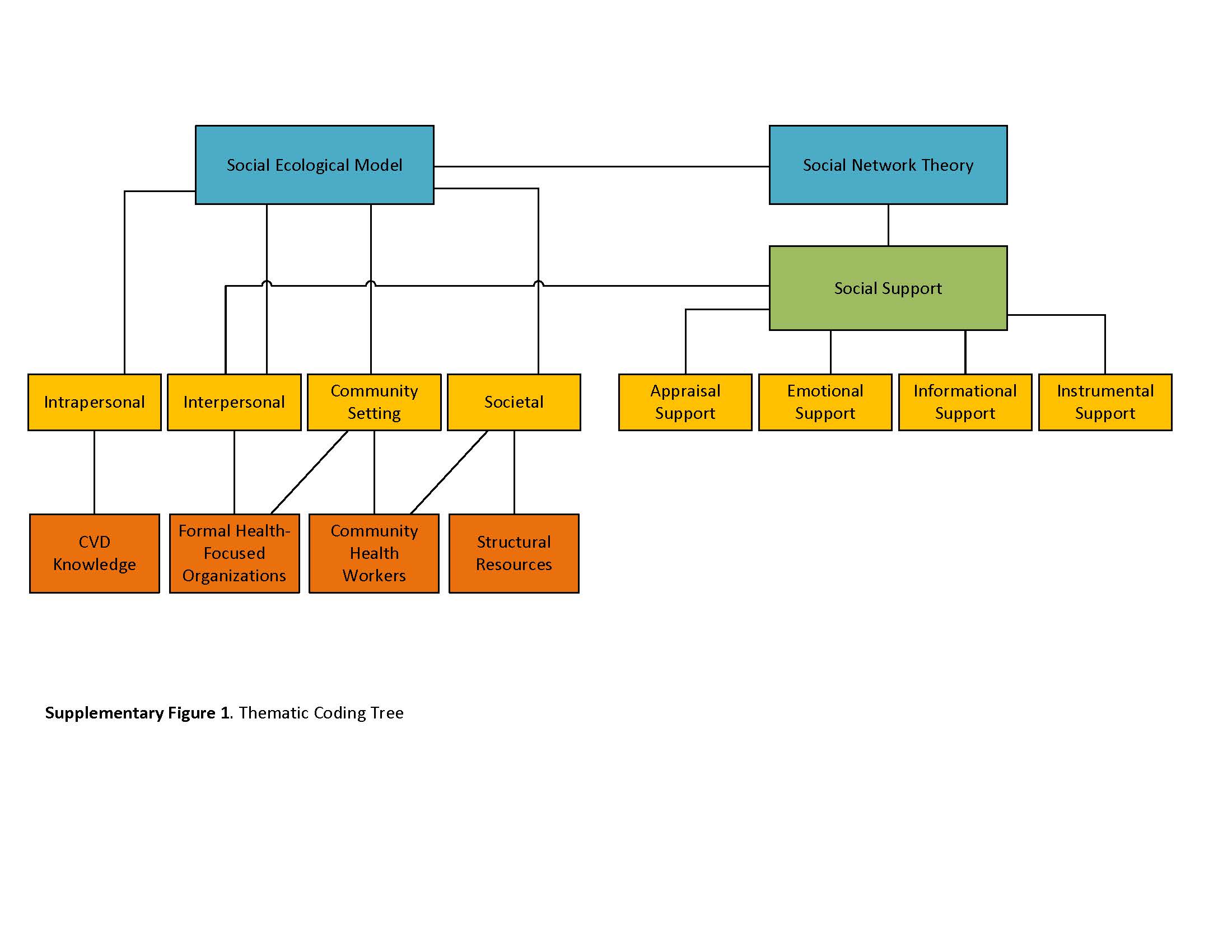

Supplement: Supplementary file 3 [file Image_1.JPEG]
